# Comparative FISH-mapping of TTAGG telomeric sequences to the chromosomes of leafcutter ants (Formicidae, Myrmicinae): is the insect canonical sequence conserved?

**DOI:** 10.3897/CompCytogen.v14i3.52726

**Published:** 2020-08-14

**Authors:** Carini Picardi Morais de Castro, Danon Clemes Cardoso, Ricardo Micolino, Maykon Passos Cristiano

**Affiliations:** 1 Departamento de Biodiversidade, Evolução e Meio Ambiente, Universidade Federal de Ouro Preto (UFOP), MG, Brazil Universidade Federal de Ouro Preto Ouro Preto Brazil; 2 Departament de Genètica, Facultat de Biologia, Universitat de Barcelona (UB), Barcelona, Spain Universidade Barcelona Barcelona Spain; 3 Programa de Pós-graduação em Genética, Universidade Federal do Paraná (UFPR), Curitiba, PR, Brazil Universidade Federal do Paraná Curitiba Brazil

**Keywords:** evolution, FISH, insects, leafcutter ants, telomere

## Abstract

Telomeric sequences are conserved across species. The most common sequence reported among insects is (TTAGG)_n_, but its universal occurrence is not a consensus because other canonical motifs have been reported. In the present study, we used fluorescence *in situ* hybridization (FISH) using telomeric probes with (TTAGG)_6_ repeats to describe the telomere composition of leafcutter ants. We performed the molecular cytogenetic characterization of six *Acromyrmex* Mayr, 1865 and one *Atta* Fabricius, 1804 species (*Acromyrmex ambiguus* (Emery, 1888), *Ac. crassispinus* (Forel, 1909), *Ac. lundii* (Guérin-Mèneville, 1838), *Ac. nigrosetosus* (Forel, 1908), *Ac. rugosus* (Smith, 1858), *Ac. subterraneus subterraneus* (Forel, 1893), and *Atta sexdens* (Linnaeus, 1758)) and described it using a karyomorphometric approach on their chromosomes. The diploid chromosome number 2n = 38 was found in all *Acromyrmex* species, and the karyotypic formulas were as follows: *Ac. ambiguus* 2K = 14M + 12SM + 8ST + 4A, *Ac. crassispinus* 2K = 12M + 20SM + 4ST + 2A, *Ac. lundii* 2K = 10M + 14SM + 10ST + 4A, *Ac. nigrosetosus* 2K = 12M + 14SM + 10ST + 2A, and *Ac. subterraneus subterraneus* 2K = 14M + 18SM + 4ST + 2A. The exact karyotypic formula was not established for *Ac. rugosus*. FISH analyses revealed the telomeric regions in all the chromosomes of the species studied in the present work were marked by the (TTAGG)_6_ sequence. These results reinforce the premise that Formicidae presents high homology between their genera for the presence of the canonical sequence (TTAGG)_n_.

## Introduction

Cytogenetic studies have been performed on more than 750 ant species, most of which describe only the chromosome number and morphology (Lorite and Palomeque 2010; Cardoso et al. 2018a). However, the cytogenetic information available so far represents less than 5% of the known ant species. Formicidae is very diverse with respect to both karyotype and species. The subfamily Myrmicinae comprises more than 400 species with established karyotypes and haploid chromosome counts varying from n = 2 to n = 35 (Cardoso et al. 2018a). Myrmicinae includes the leafcutter ants in the genera *Atta* Fabricius, 1804 to *Acromyrmex* Mayr, 1865 that occur exclusively in the Neotropical region and are extremely important herbivores in the habitats that they occupy. They cut thousands of fresh plant pieces that are transported to nests and this habit is essential for cycling soil nutrients, mainly carbon (Farji-Brener and Ghermandi 2008). In some cases, *Atta* and *Acromyrmex* are considered agricultural pests due to the economic damages caused by their habit of cutting green leaves; therefore, most studies usually focus on their ecology, geographic distribution, and population control (Loeck et al. 2003). However, both genera need a systematic revision and a complete picture of their unclear phylogenetic relationships.

The genus *Atta* includes 17 species (Bolton 2020), of which five have an established karyotype. All species present the diploid chromosome number, 2n = 22, and the karyotype formula, 2K = 18M + 4A, except for *Atta robusta* Borgmeier, 1939, which has the formula 2K = 18M + 2SM + 2ST (reviewed in Cardoso et al. 2018a). The genus *Acromyrmex* has 34 species and 29 subspecies that are currently recognized (Bolton 2020), it has the diploid chromosome number 2n = 38 and its karyotype formula is variable (Barros et al. 2016; reviewed in Cardoso et al. 2018a). The exceptions in the genus are *Acromyrmex ameliae* de Souza, Soares & Della Lucia, 2007, that has 2n = 36 (reviewed in Cardoso et al. 2018a) and *Acromyrmex striatus* (Roger, 1863) which presents 2n = 22 (Cristiano et al. 2013). The only species whose karyotype has been characterized by morphometric analyses so far is *Ac. striatus* (Cristiano et al. 2013). Such chromosomal features are essential for understanding chromosomal variants and the possible genetic barriers among phylogenetic groups (Cardoso et al. 2018b). *Ac. striatus* is a key species within the evolutionary history of leafcutter ants because molecular analyses and its karyotype establishment resulted in reclassification of *Acromyrmex* as paraphyletic. Although *Ac. striatus* shares the characteristics of both *Acromyrmex* and *Atta*, it presents peculiarities such as its karyotype formula 2K = 20M + 2SM, indicating that *Ac. striatus* should be better classified as a genus distinct from its sibling leafcutter ants (Cristiano et al. 2013).

Karyo-evolutionary pathways can be accurately established from molecular analyses by means of fluorescence *in situ* hybridization (FISH), a chromosomal mapping technique that allows identification of specific genomic regions through hybridization of fluorescent probes to the genetic material (Speicher and Carter 2005). Probe origin may range from single or repetitive sequences to large genomic sequences and probes from telomeric repeating regions are commonly applied in cytogenetic studies (Micolino et al. 2019a, b, 2020; Travenzoli et al. 2019). Telomeres are located at terminal portions of chromosomes, which are enriched with repetitive bases of adenine (A), guanine (G), and thymine (T) and the number of repeated base pairs can be extremely conserved among some taxonomic groups (Blackburn 1991; Zakian 1995). Four different telomeric sequences have been identified in Insecta, but the pentanucleotide region (TTAGG)_n_ is present in most insects (Okazaki et al. 1993; Sahara et al. 1999). Thus, it is presumed that this motif is derived from a common ancestor and is therefore homologous among the class orders (Vítková et al. 2005). However, many Hymenoptera families do not present the sequence in their chromosomes (Menezes et al. 2017), whereas some families have several species that show telomeric regions marked by the presence of (TTAGG)_n_ or the vertebrate canonical repetition (TTAGGG)_n_, as in the case of Apidae (Sahara et al. 1999), Formicidae (Okazaki et al. 1993; Meyne et al. 1995; Lorite et al. 2002; Wurm et al. 2011) and Tenthredinidae, which has two species presenting the insect canonical sequence (Gokhman and Kuznetsova 2018).

The pentanucleotide sequence has apparently evolved from the canonical sequence (TTAGGG)_n_ and has changed during insect diversification. This is supported by families that show the presence of (TTAGGG)_n_ and also by genera which present a different telomeric sequence such as (TCAGG)_n_, which is observed in some Coleoptera families (Kuznetsova et al. 2019). The differences in telomeric sequences within the class Insecta can be explained by biological mechanisms that preserve the telomere integrity. Telomerase is the enzyme responsible for maintaining repetitive sequences on telomeres; however, many alternative telomerase-independent mechanisms also act in telomere conservation. In this manner, the (TTAGG)_n_ sequence has been lost and recovered several times during the evolution of insects (Kuznetsova et al. 2019).

Other than chromosome number, not much cytogenetic information is available regarding leafcutter ants, and FISH analyses involving telomeric probes are available only for *Ac. striatus* (Pereira et al. 2018). Further, the distribution of canonical repeats and telomerase systems is still an open question among insects (Kuznetsova et al. 2019). Thus, in the present study, we analyzed the homology between the telomeric regions of leafcutter ant species *Ac. ambiguus* (Emery, 1888), *Ac. crassispinus* (Forel, 1909), *Ac. lundii* (Guérin-Mèneville, 1838), *Ac. nigrosetosus* (Forel, 1908), *Ac. rugosus* (Smith, 1858), *Ac. subterraneus subterraneus* (Forel, 1893), and *Atta sexdens* (Linnaeus, 1758) by FISH chromosome mapping using the (TTAGG)_6_ probe. We aimed to accumulate evolutionary evidence for the presence of an insect canonical telomere motif on the chromosomes of leafcutter ants. We further performed a detailed karyomorphometric analysis to establish karyotypes and classify chromosome, and described two new chromosome counts.

## Material and methods

### Chromosome preparation and fluorescence *in situ* hybridization (FISH)

The ant colonies were collected from different Brazilian states in 2018. *Acromyrmex ambiguus* was collected from Ilha Comprida – SP (24°44'28"S, 47°32'24"W); the species *Ac. crassispinus* (Ouro Preto – 20°17'15"S, 43°30'29"W), *Ac. rugosus* (Marliéria – 19°43'21"S, 42°43'26"W), *Ac. nigrosetosus* (Ouro Preto – 20°17'15"S, 43°30'29"W), *Ac. subterraneus subterraneus* (Viçosa – 20°48'35.5"S, 42°51'31.07"W), and *At. sexdens* (Marliéria – 19°43'21"S, 42°43'26"W) were collected in Minas Gerais – MG; *Ac. lundii* was collected in Dom Pedrito – RS (30°58'5"S, 54°40'23"W). The nests were kept at the Laboratório de Genética Evolutiva e de Populações of the Universidade Federal de Ouro Preto. The brain ganglia of post-defective larvae were extracted in hypotonic solution of colchicine (0.005%), as described by Imai et al. (1988) with modifications described by Cardoso et al. (2012), to obtain the metaphasic chromosomes.

FISH experiments were performed as described by Micolino et al. (2019a). The (TTAGG)_6_ motif was directly labeled with Cy3 at the 5' terminal (Sigma, St. Louis, MO, USA). Briefly, slides were submitted to RNA degradation for 1 h in a humid chamber at 37 °C, were washed in 2× SSC, and treated with 0.005% pepsin for 10 min. After washing in 1× PBS, the slides were fixed with 10% formaldehyde for 10 min. Another wash in 1× PBS was performed and then, the slides were dehydrated in an alcohol series. Chromosomal denaturation was promoted by adding 70% formamide at 75 °C for 5 min. Another alcohol dehydration series was performed before adding 2 μL of the (TTAGG)_6_ probe and 18 μL of HybMix to each slide in the dark. The slides were incubated overnight in a humid chamber at 37 °C. Finally, the slides were washed in 2× SSC solution, 1× SSC, 4× SSC Tween (during 5 min in each solution), and then rapidly in 1× PBS. Dehydration was performed in an alcohol series and DAPI was added as a counterstain. To select 10 metaphases with chromosomal integrity and evident probe marking, the slides were visualized on a Zeiss Axio Imager Z2 fluorescence microscope coupled with an image capture system and the resulting images were further edited using Adobe Photoshop CC Software.

### Karyomorphometry

The slides were stained with a 4% Giemsa solution and visualized on a Zeiss Axio Imager Z2 microscope with image capture. For each species, we selected 10 metaphases with chromosomal integrity, evident centromeres and no overlapping. Karyomorphometry and chromosomal classification were performed as described by Cristiano et al. (2017). The chromosomes were measured using Image-Pro Plus (Media Cybernetics, Rockville, MD) and some chromosome characteristics were evaluated. For each chromosome, we measured the total length (TL) end-to-end, short arm (S), and long arm (L) sizes calculated by the distance between the arm end and centromeric region. The karyotype length (KL) was calculated by summing the total length of all chromosomes. The relative size (RL) of each chromosome was calculated in relation to the total size of all chromosomes (TL × 100 / ΣTL). The ratio (r) between the length of the long arm and short arm (r = L / S) was calculated to classify the chromosomes as described by Levan et al. (1964) with modifications reported by Crozier (1970).

## Results

**Figure 1.**
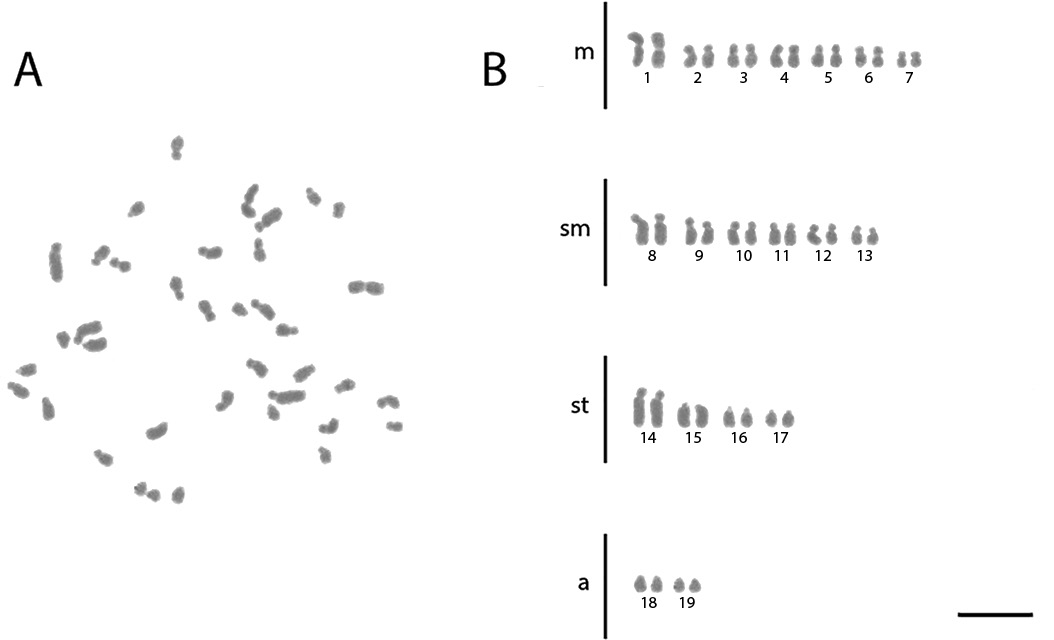
Conventional staining of mitotic cells of *Acromyrmex ambiguus***A** the metaphase and **B** diploid karyotype with 2n = 38. Scale bar: 5 µm.

**Figure 2.**
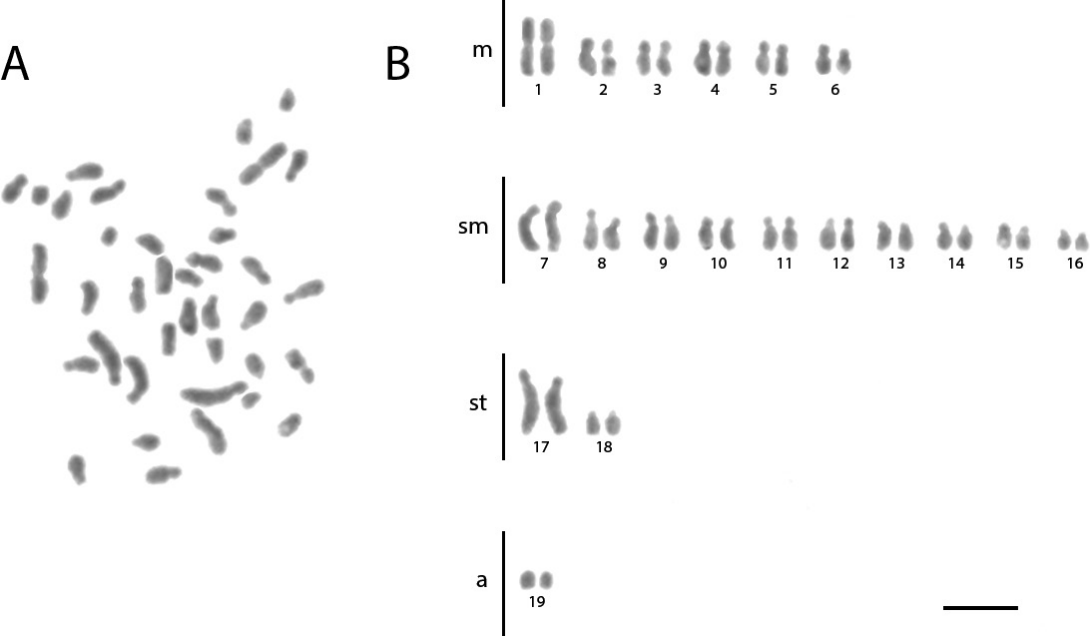
Conventional staining of mitotic cells of *Acromyrmex crassispinus***A** the metaphase and **B** diploid karyotype with 2n = 38. Scale bar: 5 µm.

**Figure 3.**
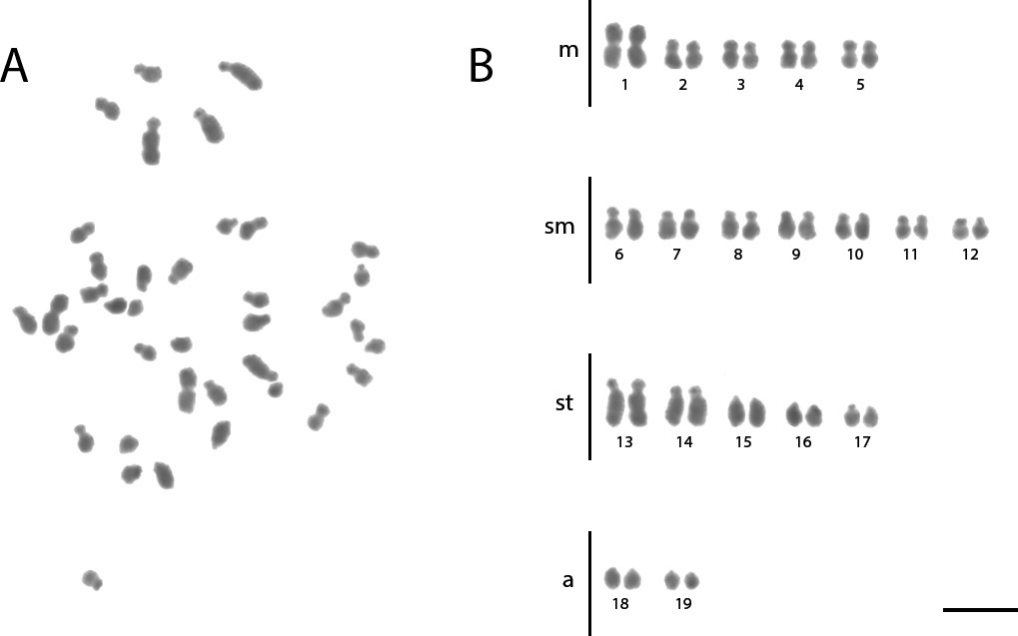
Conventional staining of mitotic cells of *Acromyrmex lundii***A** the metaphase and **B** diploid karyotype with 2n = 38. Scale bar: 5 µm.

**Figure 4.**
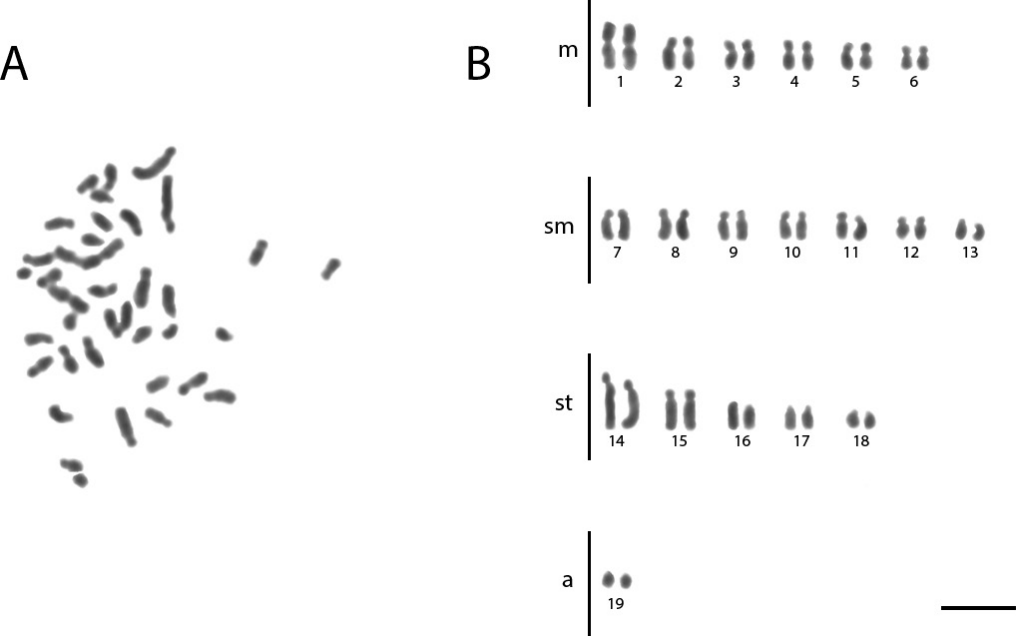
Conventional staining of mitotic cells of *Acromyrmex nigrosetosus***A** the metaphase and **B** diploid karyotype with 2n = 38. Scale bar: 5 µm.

**Figure 5.**
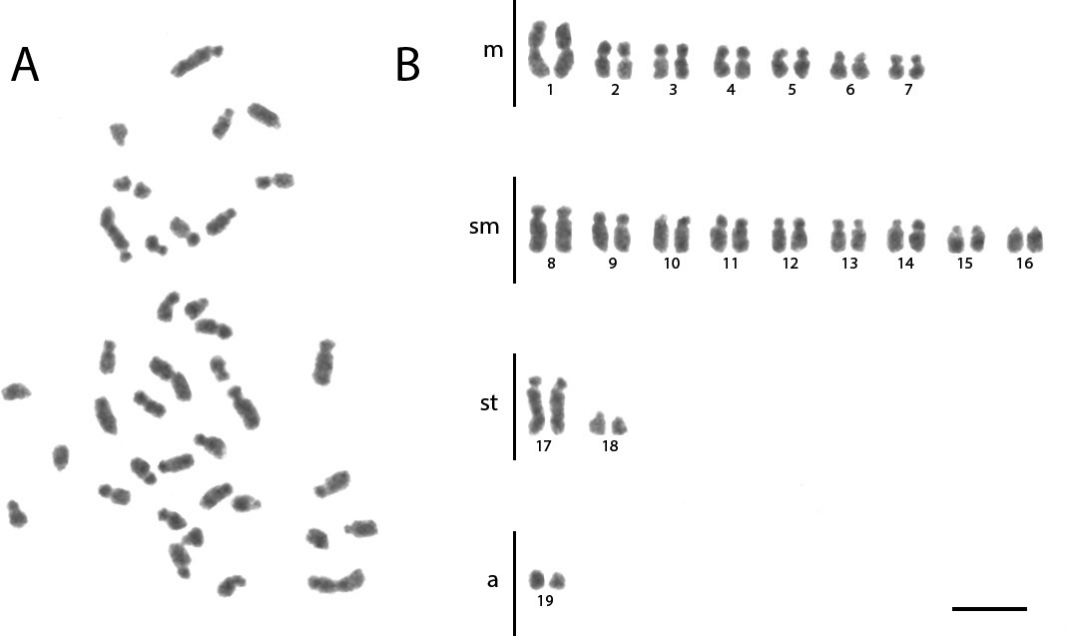
Conventional staining of mitotic cells of *Acromyrmex subterraneus subterraneus***A** the metaphase and **B** diploid karyotype with 2n = 38. Scale bar: 5 µm.

The typical chromosome number of *Acromyrmex* (2n = 38) was found in all species of the genus analyzed in the present work. The karyotype of *Ac. lundii* and *Ac. nigrosetosus* were described for the first time and, that of *Ac. ambiguus* was described for the first time from a Brazilian population. The two largest chromosomal pairs were the first subtelocentric and the first metacentric. The karyotype formula was variable (see below) and in *Ac. crassispinus*, *Ac. lundii*, *Ac. nigrosetosus*, and *Ac. subterraneus subterraneus*, most chromosomes presented an r ratio between 1.67 and 3.00; therefore, these were classified as submetacentric. The chromosomal classification of *Ac. ambiguus* was different from that of other species, as it mainly presents metacentric chromosomes. *Ac. ambiguus* has the karyotype formula 2K = 14M + 12SM + 8ST + 4A (Figure 1, Table 1). *Ac. crassispinus* presented 2K = 12M + 20SM + 4ST + 2A (Figure 2, Table 2) and its chromosomes are larger when compared to other species. *Ac. lundii* has the karyotype formula 2K = 10M + 14SM + 10ST + 4A (Figure 3, Table 3). *Ac. nigrosetosus* presented 2K = 12M + 14SM + 10ST + 2A and its chromosomes seem smaller than those of the other species (Figure 4, Table 4). *Ac. subterraneus subterraneus* has 2K = 14M + 18SM + 4ST + 2A (Figure 5, Table 5). For *Ac. rugosus* and *At. sexdens* only the chromosome number was established, but no detailed karyomorphometry was performed.

**Figure 6.**
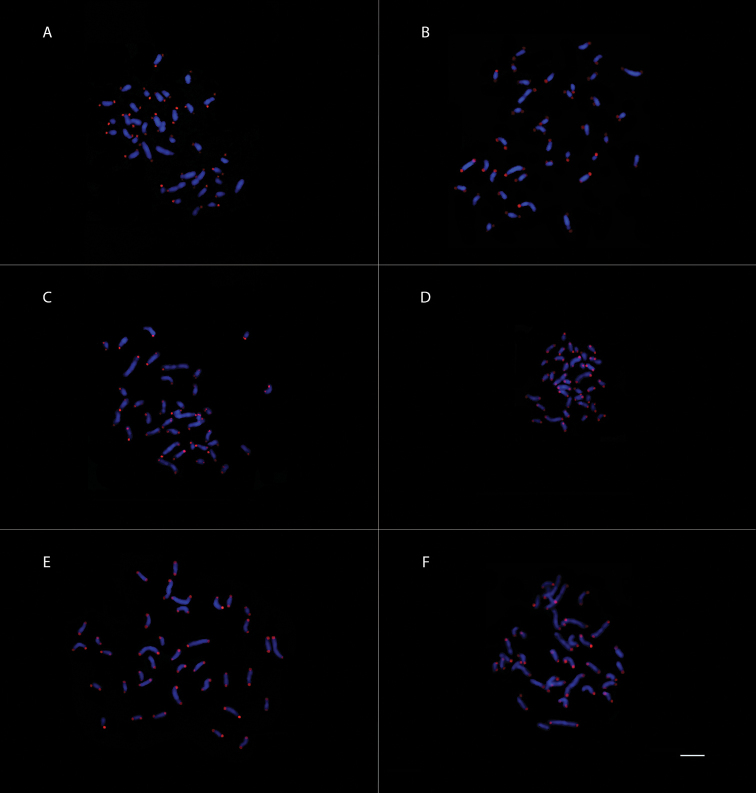
FISH mapping of mitotic metaphase chromosomes using a (TTAGG)_6_ telomeric probe; DAPI stain in blue and Cy3 in red **A***Acromyrmex ambiguus***B***Acromyrmex crassispinus***C***Acromyrmex lundii***D***Acromyrmex nigrosetosus***E***Acromyrmex rugosus* and **F***Acromyrmex subterraneus subterraneus.* Scale bar: 5 µm.

**Figure 7.**
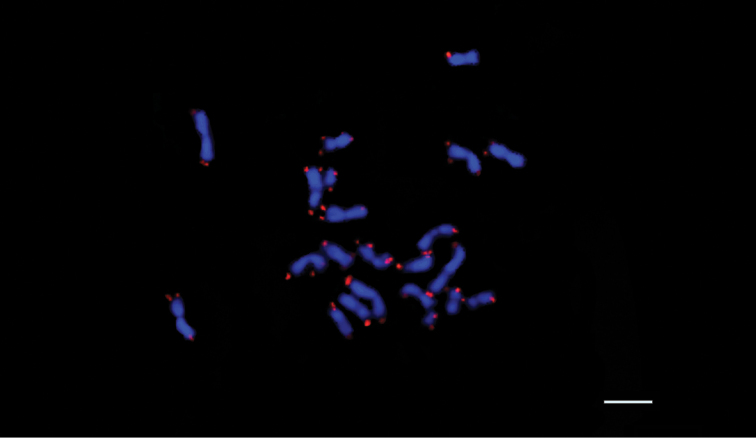
FISH mapping of *Atta sexdens* mitotic metaphase chromosomes using a (TTAGG)_6_ telomeric probe; DAPI in blue and Cy3 in red. Scale bar: 5 µm.

FISH analyses revealed that all chromosomes of all *Acromyrmex* species and *Atta sexdens* are positively marked at both arms in the telomeric regions with the presence of the canonical insect sequence (TTAGG)_6_ and no signals for interstitial telomeric sites were detected (Figures 6A–F, 7).. The intensity and size of the probe marking was varied between the chromosomes and metaphases of each species.

**Table 1.** Karyomorphometric analyses of the chromosomes of *Acromyrmex ambiguus*.

Chromosomes	TL	L	S	RL	r	Classification
**1**	5.13 ± 1.90	2.79 ± 1.03	2.34 ± 0.89	4.41 ± 0.39	1.20 ± 0.14	Metacentric
**1**	4.85 ± 1.87	2.60 ± 1.01	2.26 ± 0.89	4.17 ± 0.46	1.15 ± 0.15	Metacentric
**2**	3.35 ± 1.11	1.96 ± 0.68	1.39 ± 0.43	2.91 ± 0.14	1.40 ± 0.14	Metacentric
**2**	3.18 ± 0.98	1.87 ± 0.58	1.31 ± 0.41	2.78 ± 0.05	1.43 ± 0.14	Metacentric
**3**	3.11 ± 0.94	1.83 ± 0.59	1.29 ± 0.36	2.72 ± 0.07	1.41 ± 0.10	Metacentric
**3**	3.08 ± 0.93	1.80 ± 0.58	1.28 ± 0.36	2.69 ± 0.07	1.40 ± 0.10	Metacentric
**4**	3.01 ± 0.92	1.76 ± 0.57	1.25 ± 0.36	2.63 ± 0.06	1.40 ± 0.13	Metacentric
**4**	2.92 ± 0.89	1.77 ± 0.54	1.15 ± 0.35	2.55 ± 0.09	1.54 ± 0.10	Metacentric
**5**	2.86 ± 0.84	1.71 ± 0.52	1.15 ± 0.33	2.50 ± 0.08	1.48 ± 0.13	Metacentric
**5**	2.76 ± 0.77	1.62 ± 0.47	1.14 ± 0.32	2.43 ± 0.15	1.43 ± 0.18	Metacentric
**6**	2.65 ± 0.69	1.61 ± 0.44	1.04 ± 0.25	2.35 ± 0.18	1.54 ± 0.12	Metacentric
**6**	2.56 ± 0.66	1.47 ± 0.36	1.09 ± 0.33	2.27 ± 0.21	1.39 ± 0.18	Metacentric
**7**	2.28 ± 0.62	1.37 ± 0.40	0.90 ± 0.23	2.02 ± 0.22	1.51 ± 0.10	Metacentric
**7**	2.13 ± 0.55	1.22 ± 0.31	0.90 ± 0.26	1.89 ± 0.17	1.37 ± 0.18	Metacentric
**8**	4.35 ± 1.37	3.17 ± 1.02	1.18 ± 0.36	3.79 ± 0.18	2.68 ± 0.27	Submetacentric
**8**	4.11 ± 1.27	3.01 ± 0.95	1.10 ± 0.33	3.59 ± 0.16	2.73 ± 0.19	Submetacentric
**9**	3.35 ± 0.99	2.36 ± 0.77	0.98 ± 0.26	2.94 ± 0.13	2.40 ± 0.41	Submetacentric
**9**	3.15 ± 0.94	2.21 ± 0.76	0.94 ± 0.21	2.76 ± 0.08	2.33 ± 0.44	Submetacentric
**10**	3.11 ± 0.91	2.15 ± 0.68	0.95 ± 0.25	2.73 ± 0.09	2.25 ± 0.32	Submetacentric
**10**	3.07 ± 0.92	2.17 ± 0.73	0.90 ± 0.22	2.69 ± 0.07	2.41 ± 0.44	Submetacentric
**11**	2.98 ± 0.91	2.08 ± 0.66	0.90 ± 0.26	2.60 ± 0.08	2.33 ± 0.28	Submetacentric
**11**	2.90 ± 0.86	2.00 ± 0.63	0.90 ± 0.24	2.54 ± 0.07	2.20 ± 0.22	Submetacentric
**12**	2.70 ± 0.68	1.77 ± 0.58	0.93 ± 0.20	2.40 ± 0.22	2.08 ± 0.28	Submetacentric
**12**	2.57 ± 0.67	1.76 ± 0.45	0.81 ± 0.23	2.29 ± 0.24	2.21 ± 0.22	Submetacentric
**13**	2.47 ± 0.66	1.73 ± 0.47	0.75 ± 0.20	2.19 ± 0.23	2.33 ± 0.28	Submetacentric
**13**	2.19 ± 0.51	1.49 ± 0.39	0.70 ± 0.14	1.96 ± 0.25	2.10 ± 0.26	Submetacentric
**14**	5.22 ± 1.84	4.15 ± 1.60	1.07 ± 0.30	4.50 ± 0.32	3.86 ± 0.97	Subtelocentric
**14**	4.76 ± 1.56	3.79 ± 1.33	0.97 ± 0.26	4.14 ± 0.19	3.86 ± 0.66	Subtelocentric
**15**	3.23 ± 1.24	2.61 ± 1.03	0.62 ± 0.22	2.77 ± 0.29	4.13 ± 0.62	Subtelocentric
**15**	2.99 ± 1.15	2.35 ± 0.93	0.64 ± 0.25	2.56 ± 0.31	3.68 ± 0.65	Subtelocentric
**16**	2.69 ± 1.05	2.15 ± 0.88	0.54 ± 0.19	2.29 ± 0.25	3.98 ± 0.60	Subtelocentric
**16**	2.55 ± 0.96	1.98 ± 0.76	0.57 ± 0.21	2.18 ± 0.20	3.49 ± 0.45	Subtelocentric
**17**	2.39 ± 0.87	1.91 ± 0.73	0.48 ± 0.17	2.05 ± 0.16	4.00 ± 0.93	Subtelocentric
**17**	2.21 ± 0.76	1.74 ± 0.58	0.48 ± 0.20	1.91 ± 0.14	3.93 ± 1.11	Subtelocentric
**18**	2.03 ± 0.48	1.83 ± 0.43	0.20 ± 0.06	1.82 ± 0.22	9.14 ± 1.41	Acrocentric
**18**	1.95 ± 0.47	1.73 ± 0.41	0.22 ± 0.07	1.74 ± 0.20	8.27 ± 0.99	Acrocentric
**19**	1.85 ± 0.43	1.66 ± 0.39	0.19 ± 0.04	1.66 ± 0.19	9.02 ± 0.91	Acrocentric
**19**	1.75 ± 0.42	1.57 ± 0.39	0.18 ± 0.03	1.56 ± 0.19	8.79 ± 1.21	Acrocentric
**KL**	114.44					

TL: total length; L: long arm length; S: short arm length; RL: relative length; r: arm ratio, KL: karyotype length.

**Table 2.** Karyomorphometric analyses of the chromosomes of *Acromyrmex crassispinus*.

Chromosomes	TL	L	S	RL	r	Classification
**1**	5.84 ± 0.93	3.10 ± 0.51	2.74 ± 0.47	4.35 ± 0.22	1.14 ± 0.12	Metacentric
**1**	5.66 ± 0.93	3.04 ± 0.44	2.62 ± 0.50	4.21 ± 0.21	1.17 ± 0.08	Metacentric
**2**	3.96 ± 0.76	2.36 ± 0.53	1.61 ± 0.28	2.94 ± 0.20	1.47 ± 0.20	Metacentric
**2**	3.74 ± 0.60	2.24 ± 0.32	1.50 ± 0.31	2.79 ± 0.10	1.51 ± 0.14	Metacentric
**3**	3.63 ± 0.56	2.10 ± 0.24	1.45 ± 0.20	2.71 ± 0.10	1.46 ± 0.15	Metacentric
**3**	3.58 ± 0.56	1.98 ± 0.31	1.60 ± 0.29	2.67 ± 0.10	1.25 ± 0.14	Metacentric
**4**	3.48 ± 0.50	2.04 ± 0.34	1.43 ± 0.19	2.60 ± 0.08	1.43 ± 0.13	Metacentric
**4**	3.38 ± 0.48	2.01 ± 0.27	1.37 ± 0.23	2.53 ± 0.12	1.49 ± 0.14	Metacentric
**5**	3.23 ± 0.46	1.94 ± 0.30	1.30 ± 0.18	2.42 ± 0.15	1.50 ± 0.10	Metacentric
**5**	3.11 ± 0.49	1.85 ± 0.33	1.27 ± 0.18	2.33 ± 0.18	1.45 ± 0.13	Metacentric
**6**	2.94 ± 0.53	1.63 ± 0.42	1.14 ± 0.27	2.19 ± 0.21	1.43 ± 0.16	Metacentric
**6**	3.01 ± 1.11	1.86 ± 0.89	1.14 ± 0.31	2.21 ± 0.57	1.60 ± 0.45	Metacentric
**7**	5.02 ± 0.83	3.57 ± 0.54	1.45 ± 0.37	3.74 ± 0.12	2.53 ± 0.42	Submetacentric
**7**	4.72 ± 0.86	3.22 ± 0.90	1.50 ± 0.45	3.51 ± 0.24	2.49 ± 0.29	Submetacentric
**8**	3.99 ± 0.58	2.70 ± 0.44	1.29 ± 0.23	2.98 ± 0.12	2.14 ± 0.39	Submetacentric
**8**	3.85 ± 0.59	2.66 ± 0.40	1.20 ± 0.23	2.87 ± 0.08	2.25 ± 0.34	Submetacentric
**9**	3.78 ± 0.57	2.65 ± 0.37	1.13 ± 0.24	2.82 ± 0.08	2.39 ± 0.34	Submetacentric
**9**	3.70 ± 0.60	2.56 ± 0.50	1.14 ± 0.19	2.75 ± 0.08	2.29 ± 0.45	Submetacentric
**10**	3.64 ± 0.57	2.51 ± 0.45	1.13 ± 0.20	2.71 ± 0.07	2.25 ± 0.41	Submetacentric
**10**	3.56 ± 0.52	2.43 ± 0.35	1.12 ± 0.22	2.65 ± 0.05	2.20 ± 0.33	Submetacentric
**11**	3.48 ± 0.48	2.41 ± 0.36	1.07 ± 0.17	2.60 ± 0.08	2.27 ± 0.34	Submetacentric
**11**	3.39 ± 0.50	2.32 ± 0.40	1.07 ± 0.18	2.53 ± 0.07	2.19 ± 0.42	Submetacentric
**12**	3.34 ± 0.48	2.31 ± 0.39	1.02 ± 0.14	2.49 ± 0.09	2.27 ± 0.33	Submetacentric
**12**	3.25 ± 0.46	2.21 ± 0.39	1.05 ± 0.12	2.43 ± 0.11	2.10 ± 0.31	Submetacentric
**13**	3.15 ± 0.48	2.14 ± 0.39	1.01 ± 0.13	2.35 ± 0.12	2.13 ± 0.27	Submetacentric
**13**	2.98 ± 0.50	2.07 ± 0.40	0.92 ± 0.15	2.22 ± 0.12	2.27 ± 0.39	Submetacentric
**14**	2.84 ± 0.43	1.90 ± 0.35	0.93 ± 0.13	2.11 ± 0.06	2.06 ± 0.34	Submetacentric
**14**	2.77 ± 0.43	1.91 ± 0.33	0.86 ± 0.13	2.07 ± 0.07	2.23 ± 0.28	Submetacentric
**15**	2.71 ± 0.43	1.88 ± 0.26	0.83 ± 0.22	2.02 ± 0.09	2.34 ± 0.44	Submetacentric
**15**	2.67 ± 0.43	1.79 ± 0.30	0.87 ± 0.16	1.99 ± 0.08	2.08 ± 0.31	Submetacentric
**16**	2.55 ± 0.43	1.75 ± 0.32	0.80 ± 0.16	1.90 ± 0.11	2.24 ± 0.43	Submetacentric
**16**	2.48 ± 0.45	1.68 ± 0.32	0.80 ± 0.17	1.84 ± 0.18	2.14 ± 0.37	Submetacentric
**17**	6.43 ± 1.18	5.09 ± 0.95	1.34 ± 0.28	4.77 ± 0.20	3.83 ± 0.48	Subtelocentric
**17**	5.99 ± 0.93	4.67 ± 0.74	1.31 ± 0.20	4.46 ± 0.15	3.58 ± 0.29	Subtelocentric
**18**	2.34 ± 0.44	1.83 ± 0.35	0.51 ± 0.10	1.75 ± 0.18	3.65 ± 0.62	Subtelocentric
**18**	2.09 ± 0.43	1.68 ± 0.37	0.41 ± 0.09	1.55 ± 0.15	4.13 ± 0.76	Subtelocentric
**19**	2.03 ± 0.37	1.82 ± 0.32	0.21 ± 0.06	1.51 ± 0.13	9.02 ± 1.69	Acrocentric
**19**	1.91 ± 0.26	1.70 ± 0.23	0.20 ± 0.05	1.43 ± 0.10	8.69 ± 1.68	Acrocentric
**KL**	134.22					

TL: total length; L: long arm length; S: short arm length; RL: relative length; r: arm ratio, KL: karyotype length.

**Table 3.** Karyomorphometric analyses of the chromosomes of *Acromyrmex lundii*.

Chromosomes	TL	L	S	RL	r	Classification
**1**	5.00 ± 1.17	2.77 ± 0.70	2.23 ± 0.49	4.42 ± 0.27	1.24 ± 0.11	Metacentric
**1**	4.67 ± 1.10	2.62 ± 0.68	2.05 ± 0.44	4.14 ± 0.28	1.27 ± 0.13	Metacentric
**2**	3.16 ± 0.63	1.81 ± 0.35	1.35 ± 0.30	2.82 ± 0.08	1.35 ± 0.11	Metacentric
**2**	3.06 ± 0.66	1.79 ± 0.36	1.27 ± 0.32	2.72 ± 0.10	1.43 ± 0.16	Metacentric
**3**	2.91 ± 0.61	1.70 ± 0.38	1.21 ± 0.28	2.59 ± 0.09	1.42 ± 0.21	Metacentric
**3**	2.92 ± 0.62	1.67 ± 0.32	1.25 ± 0.32	2.60 ± 0.09	1.37 ± 0.18	Metacentric
**4**	2.81 ± 0.53	1.67 ± 0.32	1.14 ± 0.22	2.51 ± 0.08	1.47 ± 0.11	Metacentric
**4**	2.75 ± 0.51	1.63 ± 0.30	1.13 ± 0.21	2.46 ± 0.08	1.45 ± 0.09	Metacentric
**5**	2.61 ± 0.43	1.53 ± 0.28	1.07 ± 0.19	2.34 ± 0.15	1.44 ± 0.18	Metacentric
**5**	2.42 ± 0.45	1.43 ± 0.30	0.99 ± 0.16	2.17 ± 0.20	1.44 ± 0.13	Metacentric
**6**	3.38 ± 0.65	2.30 ± 0.44	1.09 ± 0.22	3.02 ± 0.15	2.12 ± 0.22	Submetacentric
**6**	3.24 ± 0.60	2.17 ± 0.42	1.08 ± 0.20	2.89 ± 0.11	2.02 ± 0.21	Submetacentric
**7**	3.19 ± 0.64	2.19 ± 0.43	1.00 ± 0.22	2.84 ± 0.12	2.20 ± 0.19	Submetacentric
**7**	3.12 ± 0.62	2.19 ± 0.48	0.93 ± 0.17	2.78 ± 0.13	2.36 ± 0.32	Submetacentric
**8**	3.02 ± 0.55	2.07 ± 0.39	0.95 ± 0.21	2.70 ± 0.13	2.21 ± 0.36	Submetacentric
**8**	2.96 ± 0.53	2.00 ± 0.39	0.96 ± 0.19	2.65 ± 0.12	2.12 ± 0.40	Submetacentric
**9**	2.88 ± 0.48	1.95 ± 0.35	0.94 ± 0.17	2.58 ± 0.14	2.10 ± 0.30	Submetacentric
**9**	2.80 ± 0.46	1.89 ± 0.32	0.91 ± 0.18	2.51 ± 0.15	2.12 ± 0.36	Submetacentric
**10**	2.70 ± 0.50	1.80 ± 0.36	0.90 ± 0.17	2.41 ± 0.12	2.02 ± 0.28	Submetacentric
**10**	2.57 ± 0.48	1.76 ± 0.32	0.82 ± 0.19	2.30 ± 0.13	2.19 ± 0.33	Submetacentric
**11**	2.40 ± 0.44	1.62 ± 0.32	0.78 ± 0.14	2.14 ± 0.11	2.07 ± 0.26	Submetacentric
**11**	2.28 ± 0.38	1.58 ± 0.34	0.70 ± 0.07	2.05 ± 0.12	2.26 ± 0.44	Submetacentric
**12**	2.18 ± 0.32	1.47 ± 0.21	0.71 ± 0.14	1.96 ± 0.14	2.10 ± 0.26	Submetacentric
**12**	2.06 ± 0.34	1.40 ± 0.26	0.67 ± 0.10	1.85 ± 0.13	2.12 ± 0.34	Submetacentric
**13**	5.01 ± 1.21	3.87 ± 0.99	1.14 ± 0.24	4.43 ± 0.24	3.38 ± 0.39	Subtelocentric
**13**	4.87 ± 1.13	3.85 ± 1.03	1.02 ± 0.12	4.31 ± 0.22	3.74 ± 0.66	Subtelocentric
**14**	4.24 ± 0.99	3.24 ± 0.79	1.00 ± 0.20	3.75 ± 0.17	3.23 ± 0.15	Subtelocentric
**14**	4.03 ± 1.00	3.08 ± 0.76	0.95 ± 0.24	3.56 ± 0.21	3.24 ± 0.17	Subtelocentric
**15**	3.22 ± 0.69	2.56 ± 0.51	0.66 ± 0.20	2.86 ± 0.19	4.00 ± 0.68	Subtelocentric
**15**	3.00 ± 0.68	2.33 ± 0.45	0.66 ± 0.25	2.66 ± 0.24	3.78 ± 0.97	Subtelocentric
**16**	2.65 ± 0.66	2.09 ± 0.49	0.56 ± 0.18	2.35 ± 0.26	3.89 ± 0.74	Subtelocentric
**16**	2.38 ± 0.51	1.85 ± 0.40	0.53 ± 0.12	2.12 ± 0.16	3.53 ± 0.26	Subtelocentric
**17**	2.27 ± 0.47	1.75 ± 0.34	0.53 ± 0.14	2.03 ± 0.14	3.40 ± 0.39	Subtelocentric
**17**	2.09 ± 0.33	1.67 ± 0.30	0.42 ± 0.08	1.88 ± 0.14	4.07 ± 0.88	Subtelocentric
**18**	2.06 ± 0.37	1.83 ± 0.33	0.23 ± 0.04	1.85 ± 0.12	7.92 ± 0.58	Acrocentric
**18**	1.93 ± 0.32	1.70 ± 0.29	0.23 ± 0.03	1.73 ± 0.14	7.58 ± 1.05	Acrocentric
**19**	1.75 ± 0.29	1.56 ± 0.25	0.18 ± 0.05	1.57 ± 0.11	8.76 ± 1.38	Acrocentric
**19**	1.64 ± 0.29	1.48 ± 0.26	0.16 ± 0.04	1.47 ± 0.07	9.09 ± 0.89	Acrocentric
**KL**	112.23					

TL: total length; L: long arm length; S: short arm length; RL: relative length; r: arm ratio, KL: karyotype length.

**Table 4.** Karyomorphometric analyses of the chromosomes of *Acromyrmex nigrosetosus*.

Chromosomes	TL	L	S	RL	r	Classification
**1**	4.40 ± 1.10	2.40 ± 0.55	2.00 ± 0.57	4.34 ± 0.34	1.22 ± 0.11	Metacentric
**1**	4.17 ± 1.00	2.24 ± 0.57	1.93 ± 0.44	4.12 ± 0.18	1.16 ± 0.08	Metacentric
**2**	2.92 ± 0.61	1.75 ± 0.33	1.18 ± 0.29	2.90 ± 0.18	1.51 ± 0.12	Metacentric
**2**	2.79 ± 0.58	1.68 ± 0.34	1.12 ± 0.24	2.77 ± 0.12	1.51 ± 0.10	Metacentric
**3**	2.71 ± 0.54	1.57 ± 0.38	1.14 ± 0.20	2.70 ± 0.10	1.38 ± 0.21	Metacentric
**3**	2.65 ± 0.53	1.61 ± 0.33	1.06 ± 0.22	2.64 ± 0.09	1.52 ± 0.14	Metacentric
**4**	2.59 ± 0.53	1.54 ± 0.34	1.04 ± 0.21	2.57 ± 0.07	1.48 ± 0.13	Metacentric
**4**	2.53 ± 0.55	1.48 ± 0.34	1.05 ± 0.22	2.50 ± 0.09	1.42 ± 0.15	Metacentric
**5**	2.44 ± 0.55	1.48 ± 0.35	0.96 ± 0.20	2.42 ± 0.12	1.54 ± 0.11	Metacentric
**5**	2.37 ± 0.55	1.42 ± 0.32	0.96 ± 0.24	2.35 ± 0.13	1.48 ± 0.09	Metacentric
**6**	2.24 ± 0.56	1.33 ± 0.35	0.90 ± 0.22	2.21 ± 0.19	1.48 ± 0.12	Metacentric
**6**	2.06 ± 0.39	1.24 ± 0.25	0.82 ± 0.14	2.06 ± 0.14	1.52 ± 0.14	Metacentric
**7**	2.99 ± 0.55	2.11 ± 0.41	0.88 ± 0.17	2.98 ± 0.18	2.42 ± 0.27	Submetacentric
**7**	2.88 ± 0.56	2.00 ± 0.42	0.88 ± 0.18	2.87 ± 0.14	2.29 ± 0.33	Submetacentric
**8**	2.77 ± 0.56	1.90 ± 0.41	0.87 ± 0.18	2.76 ± 0.09	2.21 ± 0.26	Submetacentric
**8**	2.71 ± 0.51	1.87 ± 0.32	0.84 ± 0.22	2.70 ± 0.10	2.29 ± 0.40	Submetacentric
**9**	2.69 ± 0.52	1.87 ± 0.43	0.81 ± 0.11	2.67 ± 0.09	2.30 ± 0.36	Submetacentric
**9**	2.61 ± 0.46	1.84 ± 0.34	0.77 ± 0.15	2.60 ± 0.13	2.42 ± 0.27	Submetacentric
**10**	2.58 ± 0.46	1.79 ± 0.33	0.79 ± 0.16	2.57 ± 0.12	2.29 ± 0.35	Submetacentric
**10**	2.52 ± 0.45	1.72 ± 0.35	0.81 ± 0.14	2.51 ± 0.14	2.14 ± 0.33	Submetacentric
**11**	2.43 ± 0.47	1.67 ± 0.35	0.76 ± 0.14	2.41 ± 0.15	2.20 ± 0.29	Submetacentric
**11**	2.33 ± 0.46	1.59 ± 0.33	0.74 ± 0.15	2.31 ± 0.13	2.15 ± 0.25	Submetacentric
**12**	2.24 ± 0.42	1.52 ± 0.28	0.71 ± 0.17	2.23 ± 0.11	2.20 ± 0.39	Submetacentric
**12**	2.16 ± 0.42	1.45 ± 0.32	0.70 ± 0.15	2.14 ± 0.08	2.10 ± 0.39	Submetacentric
**13**	2.04 ± 0.40	1.39 ± 0.29	0.65 ± 0.14	2.03 ± 0.20	2.19 ± 0.41	Submetacentric
**13**	1.91 ± 0.35	1.31 ± 0.25	0.60 ± 0.13	1.91 ± 0.20	2.25 ± 0.42	Submetacentric
**14**	4.69 ± 1.10	3.73 ± 0.91	0.97 ± 0.20	4.64 ± 0.20	3.85 ± 0.39	Subtelocentric
**14**	4.40 ± 0.94	3.50 ± 0.84	0.90 ± 0.14	4.36 ± 0.12	3.89 ± 0.65	Subtelocentric
**15**	3.72 ± 0.82	2.84 ± 0.64	0.88 ± 0.19	3.68 ± 0.14	3.23 ± 0.16	Subtelocentric
**15**	3.50 ± 0.84	2.67 ± 0.64	0.83 ± 0.20	3.46 ± 0.18	3.22 ± 0.23	Subtelocentric
**16**	2.61 ± 0.66	2.05 ± 0.49	0.57 ± 0.19	2.60 ± 0.43	3.76 ± 0.79	Subtelocentric
**16**	2.35 ± 0.51	1.83 ± 0.40	0.51 ± 0.13	2.34 ± 0.31	3.64 ± 0.52	Subtelocentric
**17**	2.18 ± 0.51	1.73 ± 0.38	0.45 ± 0.14	2.17 ± 0.27	3.97 ± 0.59	Subtelocentric
**17**	2.07 ± 0.51	1.63 ± 0.40	0.44 ± 0.12	2.05 ± 0.22	3.73 ± 0.59	Subtelocentric
**18**	1.84 ± 0.50	1.47 ± 0.41	0.36 ± 0.11	1.81 ± 0.16	4.11 ± 0.54	Subtelocentric
**18**	1.70 ± 0.44	1.36 ± 0.32	0.34 ± 0.12	1.68 ± 0.12	4.15 ± 0.70	Subtelocentric
**19**	1.52 ± 0.33	1.36 ± 0.31	0.16 ± 0.03	1.51 ± 0.13	8.45 ± 1.05	Acrocentric
**19**	1.42 ± 0.29	1.27 ± 0.26	0.16 ± 0.03	1.42 ± 0.12	8.19 ± 0.79	Acrocentric
**KL**	100.73					

TL: total length; L: long arm length; S: short arm length; RL: relative length; r: arm ratio, KL: karyotype length.

**Table 5.** Karyomorphometric analyses of the chromosomes of *Acromyrmex subterraneus subterraneus*.

Chromosomes	TL	L	S	RL	r	Classification
**1**	5.03 ± 0.96	2.72 ± 0.53	2.31 ± 0.46	4.42 ± 0.37	1.19 ± 0.11	Metacentric
**1**	4.78 ± 0.94	2.55 ± 0.48	2.23 ± 0.48	4.20 ± 0.38	1.15 ± 0.10	Metacentric
**2**	3.31 ± 0.64	1.88 ± 0.33	1.43 ± 0.34	2.91 ± 0.22	1.34 ± 0.18	Metacentric
**2**	3.18 ± 0.48	1.82 ± 0.29	1.37 ± 0.21	2.81 ± 0.11	1.33 ± 0.14	Metacentric
**3**	3.08 ± 0.45	1.81 ± 0.28	1.28 ± 0.20	2.72 ± 0.10	1.42 ± 0.15	Metacentric
**3**	3.01 ± 0.44	1.78 ± 0.26	1.23 ± 0.20	2.65 ± 0.09	1.46 ± 0.13	Metacentric
**4**	2.96 ± 0.46	1.77 ± 0.30	1.19 ± 0.17	2.61 ± 0.08	1.49 ± 0.11	Metacentric
**4**	2.91 ± 0.45	1.69 ± 0.28	1.22 ± 0.18	2.56 ± 0.09	1.38 ± 0.12	Metacentric
**5**	2.87 ± 0.45	1.71 ± 0.28	1.16 ± 0.19	2.53 ± 0.10	1.48 ± 0.13	Metacentric
**5**	2.80 ± 0.42	1.70 ± 0.26	1.10 ± 0.17	2.48 ± 0.11	1.54 ± 0.12	Metacentric
**6**	2.70 ± 0.42	1.57 ± 0.18	1.12 ± 0.26	2.38 ± 0.13	1.45 ± 0.21	Metacentric
**6**	2.59 ± 0.42	1.50 ± 0.22	1.09 ± 0.24	2.29 ± 0.18	1.40 ± 0.20	Metacentric
**7**	2.46 ± 0.38	1.46 ± 0.21	1.00 ± 0.20	2.18 ± 0.17	1.48 ± 0.17	Metacentric
**7**	2.33 ± 0.39	1.40 ± 0.25	0.93 ± 0.15	2.05 ± 0.15	1.51 ± 0.14	Metacentric
**8**	4.35 ± 0.99	3.12 ± 0.69	1.22 ± 0.29	3.82 ± 0.47	2.56 ± 0.26	Submetacentric
**8**	4.05 ± 0.76	2.97 ± 0.59	1.08 ± 0.18	3.56 ± 0.32	2.74 ± 0.23	Submetacentric
**9**	3.42 ± 0.50	2.35 ± 0.41	1.08 ± 0.17	3.02 ± 0.12	2.20 ± 0.39	Submetacentric
**9**	3.32 ± 0.53	2.29 ± 0.44	1.03 ± 0.18	2.92 ± 0.14	2.26 ± 0.45	Submetacentric
**10**	3.23 ± 0.53	2.30 ± 0.41	0.93 ± 0.15	2.84 ± 0.15	2.49 ± 0.34	Submetacentric
**10**	3.20 ± 0.52	2.19 ± 0.37	1.01 ± 0.19	2.82 ± 0.15	2.19 ± 0.31	Submetacentric
**11**	3.10 ± 0.45	2.12 ± 0.35	0.98 ± 0.15	2.74 ± 0.09	2.17 ± 0.30	Submetacentric
**11**	3.04 ± 0.44	2.11 ± 0.33	0.93 ± 0.13	2.68 ± 0.07	2.27 ± 0.17	Submetacentric
**12**	3.01 ± 0.44	2.10 ± 0.38	0.91 ± 0.11	2.65 ± 0.09	2.31 ± 0.36	Submetacentric
**12**	2.94 ± 0.41	2.03 ± 0.34	0.91 ± 0.13	2.60 ± 0.10	2.26 ± 0.40	Submetacentric
**13**	2.77 ± 0.40	1.92 ± 0.35	0.84 ± 0.11	2.45 ± 0.20	2.29 ± 0.41	Submetacentric
**13**	2.68 ± 0.43	1.85 ± 0.33	0.83 ± 0.10	2.37 ± 0.19	2.21 ± 0.20	Submetacentric
**14**	2.58 ± 0.38	1.80 ± 0.30	0.77 ± 0.11	2.28 ± 0.16	2.34 ± 0.30	Submetacentric
**14**	2.48 ± 0.36	1.73 ± 0.24	0.75 ± 0.16	2.20 ± 0.17	2.35 ± 0.39	Submetacentric
**15**	2.43 ± 0.35	1.61 ± 0.23	0.82 ± 0.16	2.15 ± 0.17	2.00 ± 0.32	Submetacentric
**15**	2.29 ± 0.32	1.60 ± 0.26	0.68 ± 0.08	2.03 ± 0.14	2.35 ± 0.34	Submetacentric
**16**	2.23 ± 0.30	1.54 ± 0.21	0.69 ± 0.13	1.98 ± 0.15	2.26 ± 0.35	Submetacentric
**16**	2.16 ± 0.26	1.44 ± 0.17	0.71 ± 0.11	1.91 ± 0.11	2.05 ± 0.26	Submetacentric
**17**	4.94 ± 0.77	3.84 ± 0.63	1.1 ± 0.17	4.35 ± 0.20	3.49 ± 0.29	Subtelocentric
**17**	4.76 ± 0.70	3.73 ± 0.59	1.03 ± 0.15	4.20 ± 0.14	3.64 ± 0.46	Subtelocentric
**18**	2.12 ± 0.30	1.70 ± 0.29	0.42 ± 0.05	1.87 ± 0.12	4.12 ± 0.88	Subtelocentric
**18**	1.99 ± 0.27	1.58 ± 0.24	0.41 ± 0.08	1.76 ± 0.13	4.03 ± 1.01	Subtelocentric
**19**	1.82 ± 0.28	1.63 ± 0.24	0.19 ± 0.04	1.62 ± 0.20	8.84 ± 1.31	Acrocentric
**19**	1.61 ± 0.25	1.46 ± 0.21	0.16 ± 0.03	1.42 ± 0.14	9.23 ± 1.74	Acrocentric
**KL**	114.53					

TL: total length; L: long arm length; S: short arm length; RL: relative length; r: arm ratio, KL: karyotype length.

## Discussion

The insect canonical repeat (TTAGG)_n_ has been observed in 30 species of ants using different methods (Okazaki et al. 1993; Meyne et al. 1995; Lorite et al. 2002; Wurm et al. 2011; Pereira et al. 2018), but FISH studies were mostly performed with *Myrmecia* species (Meyne et al. 1995). The only analysis involving a leafcutter ant has been performed on *Ac. striatus*, which also presents (TTAGG)_6_ labeling in the telomeres of both arms of all 22 chromosomes and does not show markings in other chromosomal regions (Pereira et al. 2018). The present study adds information about one species of *Atta* (*At. sexdens*) and six *Acromyrmex* species (*Ac. ambiguus*, *Ac. crassispinus*, *Ac. lundii*, *Ac. nigrosetosus*, *Ac. rugosus*, *Ac. subterraneus subterraneus*). We also describe the chromosome number and structure of *Ac. lundii* and *Ac. nigrosetosus* for the first time. The karyotype description for *Ac. ambiguus* from Brazil revealed the same diploid chromosome number as in previous data available from Uruguay (Goñi 1983), but distinct regarding the karyotype formula, overrepresented by subtelocentric and acrocentric chromosomes in the latter. These differences may be due the visual determination of chromosome morphology instead chromosome measurements applied here. The new chromosome counts reported in this study again corroborate the stable chromosomal number in *Acromyrmex* and the detailed karyomorphometry of the chromosomes suggests dynamism of chromosome morphology due to distinct karyotypic formulas.

Our FISH results add to the cytogenetic knowledge of new karyotypes and molecular cytogenetic analyses in leafcutter ants, and demonstrate that the pattern found in *Ac. striatus* seems to occur in *Atta* species and *Acromyrmex* species. Importantly, *Ac. striatus* is the sister clade of *Atta* and the remaining *Acromyrmex* species (Cristiano et al. 2013). The occurrence of telomeric regions marked positively by (TTAGG)_n_ reinforces the premise that Formicidae presents high homology for the presence of the insect canonical sequence. This motif has been proposed to be a plesiomorphic chromosomal feature in Hymenoptera (Gokhman and Kuznetsova 2018). In fact, the canonical motif (TTAGG)_n_ was observed in several branches of the clade of fungus-farming ants, from anciently diverged lineages such as *Mycetophylax* to recent lineages such as *Mycetomoellerius* (Micolino et al. 2019a, b, 2020). Besides, the alternative TCAGG motif present in insects seems to be restricted to some groups, but not to Formicidae (Kuznetsova et al. 2019), and we did not find any evidences for this in previously attempted experiments in our laboratory on the phylogenetic basis of fungus-farming ants (unpublished data).

Sahara et al. (1999) propose that (TTAGG)_n_ is a sequence with high homology in Insecta because it is inherited from a common primitive ancestor of the class and the fact that some families do not show the presence of canonical repetition is explained by the group evolutionary process, where (TTAGG)_n_ has been lost and recovered several times. This theory is supported by Frydrychová et al. (2004) who studied 22 insect species from 20 different orders selected among the main phylogenetic group lineages and found that 15 species presented the (TTAGG)_n_ on their telomeres, whereas only seven species did not have the sequence in their chromosomes. The authors compared their results with the available literature and concluded that 16 insect orders have the primitive telomeric region conserved and eight do not present it. In contrast, Menezes et al. (2017) evaluated the presence of the canonical repeats (TTAGG)_n_ and (TTAGGG)_n_ in 25 representative species of eight Hymenoptera families, and surprisingly none of them showed any signs of these repetitive sequences in their telomeres or in any chromosomal regions. Therefore, the hypothesis regarding multiple losses of the sequence inherited from a primitive ancestor appears unlikely to these authors, as the number of Insecta families without the (TTAGG)_n_ sequence is higher than the number of those bearing it. Thus, the authors propose that the most probable evolutionary scenario is that the canonical repetition has been lost in the Apocrita ancestor or even in the Hymenoptera ancestor, whereas Apidae and Formicidae have recovered the region independently. On the contrary, the phylogenetic position and the presence of (TTAGG)_n_ as the telomeric repeat in *Tenthredo omissa* (Förster, 1844) and *Taxonus agrorum* (Fallén, 1808) (Tenthredinidae: Symphyta) were suggested to be indicative of the ancestrality of this motif in Hymenoptera (Gokhman and Kuznetsova 2018).

Ants have high variability in their karyotypes; there are species with the haploid number of chromosomes n = 1 (Crosland and Crozier 1986; Taylor 1991) and species with n = 60 (Mariano et al. 2008). This variation exists with respect to the chromosome number as well as the morphology and classification. Robertsonian fissions result in two acrocentric chromosomes due to the breaking of a bi-armed chromosome, whereas Robertsonian fusions involve exactly the opposite process, where two acrocentric chromosomes unite to form a single bi-armed chromosome (Lorite and Palomeque 2010). These are possibly the two most important rearrangements for karyotype evolution in ants and support the minimum-interaction theory proposed by Imai et al. (1988, 1994, 2001). This theory defines that fission processes are more significant and common than fusion processes because higher chromosome numbers reduce the possibility of interaction between non-homologous chromosomes within the nucleus, minimizing the mutation rates. Thus, it is proposed that the chromosomal number of ant species usually tends to increase. In this sense, it is also proposed that the ancestral karyotype of ants would be composed of a small number of metacentric chromosomes whereas recently divergent lineages would have more chromosomes due to several chromosomal fission processes (Imai et al. 1977). Thus, it is plausible to state that in *Acromyrmex*, karyotypes with 38 chromosomes arose following several Robertsonian fissions, whereas the chromosome number of the iconic *Ac. striatus* is a plesiomorphic feature maintained in *Atta* spp. (Cristiano et al. 2013).

Establishment of the karyotype (the chromosome number and determination of their morphology) is very important for the knowledge of chromosomal variations and possible genetic barriers between phylogenetic groups (Cristiano et al. 2017; Cardoso et al. 2018b). It is necessary to go further in describing the chromosome number and morphology, as more detailed karyomorphometric analyses may reveal additional and substantial variations not observed previously, mainly when accompanied with genome size estimates (Cardoso et al. 2018b). Tsutsui et al. (2008) state that closely related species, belonging to the same genus, may have very similar genome sizes, corroborating the pattern revealed by our karyomorphometric analyses in the *Acromyrmex* species studied here.
